# Genetic Variation in the Prion Protein Gene (*PRNP*) of Two Tunisian Goat Populations

**DOI:** 10.3390/ani11061635

**Published:** 2021-05-31

**Authors:** Samia Kdidi, Mohamed Habib Yahyaoui, Michela Conte, Barbara Chiappini, Mohamed Hammadi, Touhami Khorchani, Gabriele Vaccari

**Affiliations:** 1 Livestock and Wildlife Laboratory, Institut des Régions Arides, Université de Gabes, Route. El Djorf, Km 22.5, Medenine 4119, Tunisia; mhyhabboub@yahoo.fr (M.H.Y.); mhammadi70@gmail.com (M.H.); touha2009@gmail.com (T.K.); 2 Department of Food Safety, Nutrition and Veterinary Public Health, Istituto Superiore di Sanità, Viale Regina Elena, 299, 00161 Rome, Italy; michela.conte@iss.it (M.C.); barbara.chiappini@iss.it (B.C.); gabriele.vaccari@iss.it (G.V.)

**Keywords:** *PRNP*, polymorphism, goat, South-East, Tunisia

## Abstract

**Simple Summary:**

Goat production is contributing to the economic and social development of rural areas in arid lands, within harsh conditions of Southern Tunisia. In this geographic zone, there are two caprine populations: the native goat population and the crossed goat population. Genotyping goats for the prion protein gene (*PRNP*) allows us to estimate their level of genetic susceptibility to scrapie disease. In the present work, the Sanger sequencing method of the entire *PRNP* coding sequence was used to determine the different *PRNP* genotypes and haplotypes in two populations (116 animals). This study represents the first investigation on goats’ *PRNP* genetic variability in Tunisia, and the results are useful in the design of national breeding programs.

**Abstract:**

Scrapie is a fatal prion disease. It belongs to transmissible spongiform encephalopathies (TSEs), and occurs in sheep and goats. Similarly, to ovine species, the prion protein gene (*PRNP*) plays a major role in conferring resistance or susceptibility to TSE in goats. This study assesses the variability of *PRNP* in native and crossed-breed goat populations raised in the Southeast of Tunisia and provides information on the distribution of *PRNP* haplotypes and genotypes in these goat populations. A total of 116 unrelated goats including 82 native and 34 crossed-breed goats were screened for *PRNP* polymorphisms using Sanger sequencing. Sequence analysis revealed 10 non-synonymous polymorphisms (G37V, M137I, R139S, I142M, H143R, N146D, R154H, R211Q, Q222K, and S240P), giving rise to 12 haplotypes and 23 genotypes. Moreover, four silent mutations were detected at codons 30, 42, 138, and 179; the former was reported for the first time in goat (nucleotide 60 c→t). Interestingly, the PrP variants associated with resistance (D146 and K222) or with a prolonged incubation time of goat to scrapie (M142, R143, H154, Q211) were absent or detected with low frequencies except for H154 variant, which is present with high frequency (1%, 1%, 4%, 0%, 88%, and 6%, respectively, for native goats, and 0%, 1%, 0%, 1%, 78%, and 1%, respectively, for crossed goats). The analysis of *PRNP* polymorphisms of goats raised in other regions of the country will be useful in getting a global view of *PRNP* genetic variability and the feasibility of goat breeding programs in Tunisia.

## 1. Introduction

The transmissible spongiform encephalopathies (TSEs) or prion diseases occur in several mammal species such as humans (Creutzfeldt–Jakob disease or CJD), cattle (bovine spongiform encephalopathy or BSE) and small ruminants (scrapie of sheep and goats). These are fatal neurodegenerative disorders characterized by an accumulation of a pathological form (PrP^Sc^) of the host cellular prion protein (PrP^C^), mainly in the central nervous system [1].

The PrP gene (*PRNP*) encodes the prion protein and has been implicated in the susceptibility of sheep [2] and goat [3] to scrapie. The caprine and ovine *PRNP* is positioned in chromosome 13 [4]. Its functional length is about 21 kb with three exons. The third exon contains the whole open reading frame. The amino acid sequence of wild-type PrP^C^ seems to be the same in goat and sheep with the exception of codon 240, which can code for serine or proline in goat.

Goat *PRNP* displays a relatively high level of variability. So far, a total of 55 non-synonymous polymorphisms have been recorded [3,5,6,7,8,9,10,11,12,13] in addition to 23 silent mutations [3,8,14,15,16,17,18,19,20,21].

Similar to sheep, some caprine *PRNP* codon variants (G32stop, G127S, I142M, H143R, N146S/D, R154H, R211Q, and Q222K) were associated with different levels of resistance to TSE [3,5,6,7,8,14,15,17,18,19,22,23,24,25,26,27,28,29,30,31,32,33,34,35,36,37]. However, the most promising results have been obtained for variant K222, which was reported as conferring resistance in Italian goats [17,18]. Moreover, S146 or D146 variants have been linked to high resistance in Cyprus [19]. For these latest variants, additional studies evidenced the protection given by the presence of this allele against oral or intracerebral scrapie infection [6,28,29,30,31,32,37].

Goat production has contributed to the economic and social development of rural areas in arid lands and within harsh conditions of Southern Tunisia. In this zone, where half of the national number of goats were raised [38], goat farming provides kids’ meat and milk for family consumption. This meat production is contributing efficiently to the farmers’ income. In the oasis, where an autochthonous goat population and its crossbreeds with French Alpine and with Damasquine of Cyprus are raised, this livestock is holding to the traditional farming system and is characterized by low milk production per goat. Mixed stock farming of goats and sheep is a common practice in the Southeast of Tunisia. However, in the extensive pastoral mode, flocks of goats are complemented with sheep farming and, occasionally, with camels. In this regard, Goldmann [2] reported that breeding mixed sheep/goats in the same holdings is a possible risk factor for scrapie in goats. However, no scrapie cases have been detected so far in Tunisia. Moreover, this disease is not considered a priority and a specific program of surveillance remains absent [39] This makes it impracticable to affirm the presence of the disease in the country.

The present study aimed to determine the variability of *PRNP* and the presence of resistant haplotypes of Tunisian purebred native goat population (NGP) and its crossed goat population (CGP) raised in the southeast of the country for developing or acquiring scrapie.

## 2. Materials and Methods

### 2.1. Sample Collection

**Figure 1:**
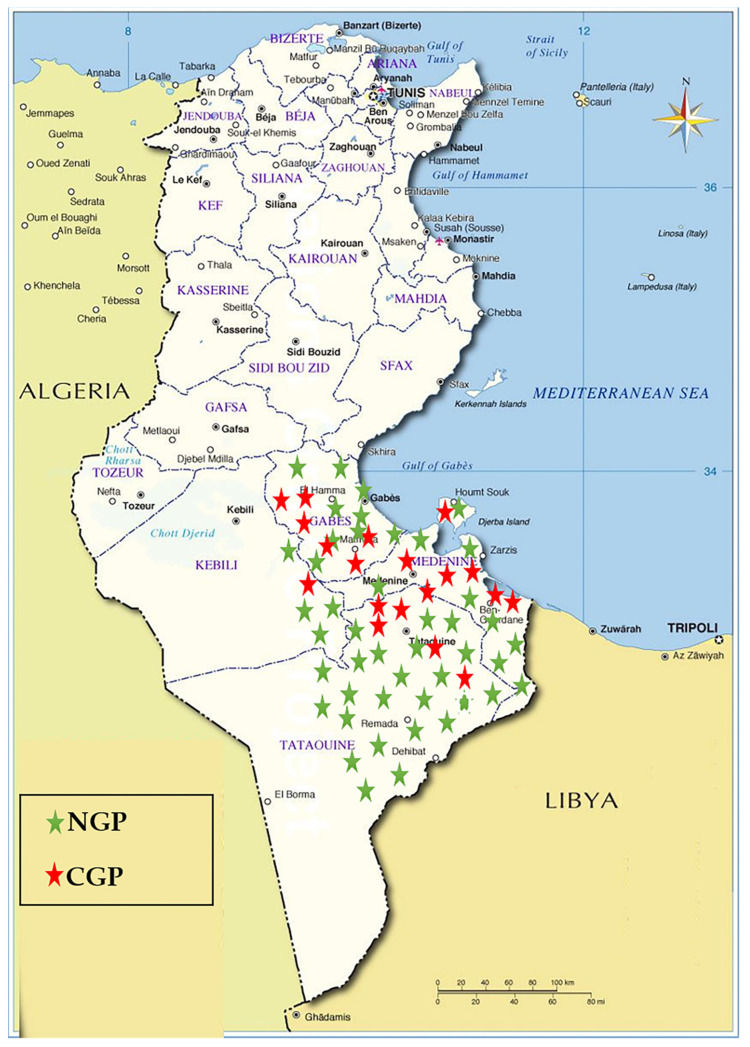
Geographical distribution of collected samples. NGP: native goat population; CGP: crossed goat population.

**Figure 2:**
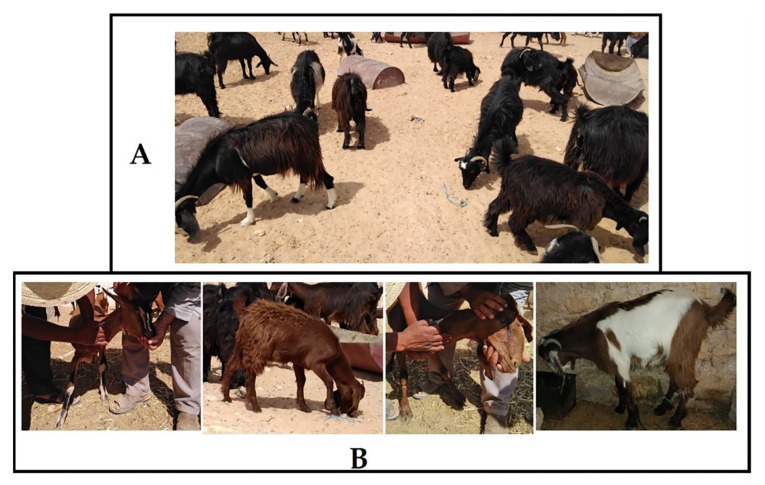
Some phenotypic profiles of NGP (**A**) and CGP (**B**) raised in Southern Tunisia.

Blood samples were collected randomly from 116 goats from goat populations in the Southeast of Tunisia (Figure 1). Sampling was carried out between the years 2007 and 2012 on clinically healthy goats belonging to NGP (*n* = 82) and CGP (*n* = 34) of Tunisia. The majority of the animals of CGP were from the local population crossbred with the Alpine French breed. The rest were the result of the cross of NGP with the Cyprus Damascus breed. Some phenotypic profiles of NGP and CGP raised in the sample collection zone are shown in Figure 2. DNA was extracted using the standard phenol–chloroform method [40].

### 2.2. Genotyping

PCR amplification and sequencing were carried out as previously reported [41]. Briefly, primers were designed over the available sequence (GenBank accession number EU032305.1) for the amplification of the entire *PRNP* coding sequence (CDS). The PCR reaction included 1× Gold Buffer and 5 units of AmpliTaq Gold (Applied Biosystems, Foster City, CA, USA), 2.5 mM MgCl_2_, 200 µM dNTPs, 0.25 µM of F1 (5′-CATTTATGACCTAGAATGTTTATAGCTGATGCCA-3′) and R1 (5′-TTGAATGAATATTATGTGGCCTCCTTCCAGAC-3′) primers and 50 ng of DNA. The PCR thermal profile started with an initial denaturation step at 94 °C (3 min) followed by 35 cycles of 30 s at 95 °C for DNA denaturation, 30 s for primer annealing at 57 °C and 1 min 30 s at 72 °C for primer extension, with a final extension step at 72 °C for 7 min. Sequencing reactions were performed with the primers T3 (5′-TTTACGTGGGCATATGATGC-3′) and T4 (5′-GGCTGCAGGTAGACACTCC-3′) using a BigDye Terminator Cycle sequencing kit v1.1 and an ABI PRISM 3130 apparatus (Applied Biosystems, Foster City, CA, USA). The sequences obtained have been aligned using Seq Scape v2.5.

### 2.3. Statistical Analysis

Allelic frequencies in the codons 37, 137, 139, 142, 143, 146, 154, 211, 222 and 240, within populations, were estimated using Arlequin program v 3.5 [42]. The same program was used to identify the haplotypes based on polymorphisms occurring at all codons for each sample. Moreover, the haplotype and genotype frequencies were estimated by direct counting.

The Hardy–Weinberg equilibrium at each codon in the two populations was assessed using the locus-by-locus test type (number of steps in the Markov chain = 1,000,000; number of dememorization steps = 100,000).

Moreover, calculation of chi-square (***χ***^2^) was used to test for the deviation from the Hardy–Weinberg equilibrium in each studied population (*p* ≤ 0.05):

(1) $$\chi^{2}=\Sigma \;({\left(O-E\right)}^{2}/E).$$

In the equation, O is the frequency of the observed genotypes, and E is the frequency of the expected genotypes.

Furthermore, the Reynolds (1983) [43] genetic distance was used in Populations 1.2.32 software [44] to compute the genetic distance between the NPG and CPG goat populations in the present work. The pairwise fixation index (F_ST_) of the analyzed populations was assessed using the Arlequin program v 3.5 [42]. This index is a measure of population differentiation, which occurs as a result of genetic structure in a population/breed.

## 3. Results

Overall, 10 non-synonymous polymorphisms within the *PRNP* CDS were identified at codons 37, 137, 139, 142, 143, 146, 154, 211, 222, and 240 (Table 1). These polymorphisms resulted in 12 estimated haplotypes (Hp) (Table 2). The allele frequencies reported in Table 1 ranged from 0 to 1. The frequencies of P and S amino acids at codon 240 seem to be similar for the two analyzed populations. Moreover, similar allele frequency values were observed at codon 154 for the two populations. For all codons, the two studied populations were in Hardy–Weinberg equilibrium.

In addition, four synonymous polymorphisms were detected, one reported here for the first time at nucleotide positions 60 (C→T), and the other three previously reported at nucleotides 126, 414, and 537.

The frequency distributions of the recorded genotypes in the two studied populations are shown in Table 3. A total of 23 genotypes were detected in the whole sample; 16 genotypes were observed for each breed. Only 9 genotypes were observed in both goat populations. The most common genotype was S240P, with a frequency of 24.5% for NGP and 21% for CGP. The genotypes S240S and P240P displayed the same frequency (15%) in the CGP and ranked the second-most prevalent genotype in this population.

The number of haplotypes found in each goat population was 9 and 10 for NGP and CGP, respectively. Seven haplotypes (Hp1, Hp3, Hp8, Hp9, Hp10, Hp11, Hp12) occurred in both the populations sampled (Table 2). Hp1, Hp9, and Hp12 were the most frequent haplotypes in NGP; however, Hp1, Hp8, and Hp12 were the most prevalent in CGP. Hp1 and Hp12 (the wild-type haplotype carrying S or P at codon 240) frequency values were not close in the studied populations. Hp9 frequency (haplotype carrying H at codon 154) was more frequent in NGP (19%) than in CGP (1%). Resistant haplotype Hp11 (carrying K222) was observed in both populations with a similar frequency (1%), while Hp7 (carrying D146) was observed only in the CGP breed (frequency 1%). Haplotypes Hp6 and Hp4 were observed only in the NGP group and haplotypes Hp7, Hp5, and Hp2 only in the CGP group, all with a frequency ranging between 1% and 4%.

The Hardy–Weinberg equilibrium was holding for the two populations (*p* > 0.05). Table 4 shows similar values for the Reynolds distance (DR) and pairwise FST differences. The two values were low and highlighted a very low genetic differentiation between NGP and CGP, and thus the studied populations had small genetic distance.

## 4. Discussion

In the present study, we determined the *PRNP* polymorphisms of native and crossed goat populations, which are the only two goat populations raised in the Southeast of Tunisia in order to evaluate the presence of polymorphisms associated with scrapie resistance. The studied populations shared several polymorphisms and silent mutations for the same haplotypes and genotypes. The difference in the frequencies of the haplotypes and genotypes between the two populations could be attributed to the relatively small number of individuals analyzed in the CGP as compared with the NGP.

The most frequent genetic variations were in H154 (88% and 78%, respectively, for NGP and CGP) and in S240 (47% and 48%, respectively, for NGP and CGP). The allele H154 was absent in some Italian goat breeds (Facciuta della Valnerina, Fulva del Lazio, Teramana, Alpine, and Saanen) or present with very low frequencies for the rest of the breeds that have been analyzed by Torricelli et al. [45]. Some variations in other codons were absent, or were present with a low frequency, in the goat populations of the present work. The allele V37 was absent in all Algerian goat breeds [46], in Tanzanian goat breeds [9], in Ethiopian indigenous goat breeds [13], and in CGP. However, NGP showed a very low frequency of V37 that did not exceed 1%.

The most frequent genotype in the studied populations of the present work was S240P. The frequencies of this genotype were the highest also for the four Algerian goat breeds [46]. However, the frequency values of this genotype were zero in the three Turkish goat breeds (Anatolian Black, Angora, and Kilis) [4] and in the two Italian goat breeds (Cilentana and Aspromontana) [46]. For the cited Turkish and Aspromontana Italian goat breeds, the frequencies of P240P were the highest [46,47].

Haplotypes Hp1 and Hp12 were the most common in these populations. High frequencies of these haplotypes have been observed in goats from Italy, France, UK, Spain, Greece, the Netherlands, Norway, Cyprus, Tanzania, Morocco, Japan, USA, China, Algeria, and Ethiopia [9,12,20,46,47,48,49] and also in Pakistani goat breeds, which were raised near the domestication center [50].

Some *PRNP* polymorphisms have been associated with different levels of resistance to scrapie. However, neither of them is linked to full resistance. These polymorphisms were G32stop, G127S, I142M, H143R, N146S/D, R154H, R211Q, and Q222K [3,5,6,7,8,14,17,18,19,20,21,22,23,25,26,27,28,29,30,31,32,33,34,35,36,37,51].

Dassanayake et al. [32] proved that goats with the heterozygous (G/S 127) genotype have an extended incubation period compared with goats homozygous for G127 (G/G 127) following classical scrapie inoculation. This genotype was absent in the individual goats analyzed in the present work, in Small East African and Norwegian white goats [9] and in the Algerian and Italian goat breeds studied by Fantazi et al. [46], except for Aspromontana Italian goats.

The I142M polymorphism could extend the incubation period in goats [15]. In our study, the frequency of M142 does not exceed 4% in CGP, and a similar frequency value was observed in French Alpine bucks [22]. Lower frequency values were observed in three Algerian goat breeds (Naine de Kabylie, Mekatia, M’zabite) and the Italian Cilentana goat breed. These values ranged between 1.6 and 2.3% [46]. On the other hand, neither sample of NGP showed this variation. Moreover, this polymorphism was absent in Small East African goats [9].

According to Papasavva-Stylianou et al. [19], goats heterozygous for serine (S) and aspartate (D) at codon 146 were resistant to scrapie. In the present study, there were heterozygous goats for D146, but their frequency was too low and detected only in CGP. Table 2 and Table 3 show that Hp7 had two variations: D and Q in codon 146 and 211, respectively, giving rise to a heterozygous genotype that has never been defined. These results were the most probable genotype provided by the Arlequin program.

White et al. [6] tried to demonstrate whether S146 confers resistance to disease by infecting goats with an experimental challenge, and they concluded that this polymorphism in a heterozygous form means lengthening of incubation period. This alteration was absent in the whole studied sample of the current work, but present with very low frequencies in some Turkish goat breeds [47]. The frequencies were higher in another Turkish goat breed, as shown by Meydan et al. [11]. Regarding African goats, S146 was absent in Algerian goat breeds. However, in other studied goat breeds raised in Tanzania and Ethiopia, it was present with a frequency that ranged between 20% and 60% [9,13].

In its heterozygous form, the polymorphism H143R is reported to be associated with reduced scrapie susceptibility [18] in goats. In our work, this genotype was present in NGP with a low frequency. This variant was absent in Tanzanian, Ethiopian, and most of the Algerian goats analyzed, except the Mekatia breed, for which the frequency did not exceed 23% [9,13,46].

Barillet et al. [22] revealed a lower susceptibility to the disease for the R211Q heterozygous goats. In NGP and CGP, the frequencies of R211 heterozygote goats ranged between 2.75% and 5.75% in CGP but did not exceed 1.25% in NGP. This polymorphism was absent in Tanzanian and Ethiopian goat breeds [9,12] but present in only two breeds from Algeria with a lower frequency [46].

The experiment conducted by White et al. [6] and Acutis et al. [37] provided further support for previous results obtained by Goldmann et al. [14] regarding the association between K222 and the length of the classical scrapie incubation period. Moreover, Aguilar-Calvo et al. [29] investigated its effect on oral bovine spongiform encephalopathy (BSE) transmission to goats and underlined the pivotal protective effect of the K222 *PRNP* variant. In both the analyzed populations (NGP and CGP), K222 was present in heterozygous form with low frequencies. The Q222K (S240S) genotype was present with a low frequency only in CGP; this genotype was observed in Algerian M’zabite (6.45%) and Italian Cilentana goat (16.33%) breeds [46]. Another genotype combination containing Q222K (Q222K; S240P) was present only in NGP (frequency: 1.25%). Comparing this frequency with that of Moroccan goat breeds, we remark that the D’men showed the same frequency but the Chaouni showed a similar frequency [51]. Higher values were shown in the M’zabite (6.45%) and in two Italian goats (Cilentana (8.16%) and Aspromontana (16.39%)) breeds [46]. Q222K was absent in Pakistani and some Small East African caprine breeds [9,10].

The analysis by Barillet et al. [22] revealed that no PrP^Sc^-positive case was found in the R154 H heterozygous goats. This variant corresponds in our case to Hp2, Hp8, and Hp9. Furthermore, Hp2 showed the occurrence of the G37V polymorphism, and it has been shown in CGP. The G37V variant was absent in the Algerian and Italian goat breeds investigated by Fantazi et al. [46]. In other Italian goat breeds, this polymorphism was registered but with the H/H genotype [17]. Furthermore, Vaccari et al. [18] and Acutis et al. [48] found the G37V with R/R genotype in different Italian goat breeds.

On the other hand, the H154 Q222 S240 haplotype (Hp9) is a risk factor for Nor98 scrapie in goats [52]. This haplotype was detected in the two populations under study: NGP (19%) and CGP (1%). Besides, Colussi et al. [52] suggested that histidine at codon 154 of the *PRNP* gene is a risk factor for Nor98 scrapie in goats; thus, haplotypes Hp2 and Hp8 are recognized to be a risk factor for atypical scrapie.

Four silent mutations at positions 60 (c→t), 126 (g→a), 414 (c→t), and 537 (g→t) were recorded in the populations NGP and CGP. Two variants of these were detected in goats for the first time. The first new silent variation was observed at position 537 in only three animals of NGP and was shown with only the silent mutation at codon 126, whereas the second was recorded at codon 60 in only one sample of the CGP. The silent nucleotide mutation 126 was the most common in each of the populations investigated in the present work. The silent mutations at positions 126 and 414 were reported also with medium to high frequencies in Chinese goat breeds [16].

## 5. Conclusions

Our work investigated the genetic polymorphism of the *PRNP* gene in two goat populations in the Southeast of Tunisia. The non-synonymous polymorphisms concerned 10 codons, whereas only 4 silent mutations were detected in the whole sample. The two studied populations shared the most variations. The mutations associated with different levels of resistance to scrapie in goats were observed in the current study with low frequencies. The analysis of the genetic polymorphism of goat populations raised in the other geographical regions of the country seems to be of importance. It will allow getting more information on the most prevalent haplotypes and genotypes in goat populations belonging to different bioclimatic zones of Tunisia.

## Figures and Tables

**Table 1:** Estimates of allele frequencies using Arlequin v 3.5 in the studied goat populations. *n*: number of animals, NGP: native goat population, CGP: crossed goat population, AA: amino acid.

Codon Position	AA	Code of AA	Goat Populations	
			NGP (*n* = 82)	CGP (*n* = 34)
37	Glycine	G	0.99	1
	Valine	V	0.01	0
137	Methionine	M	0.97	0.96
	Isoleucine	I	0.03	0.04
139	Arginine	R	1	0.99
	Serine	S	0	0.01
142	Isoleucine	I	0.96	1
	Methionine	M	0.04	0
143	Histidine	H	1	0.99
	Arginine	R	0	0.01
146	Asparagine	N	0.99	1
	Aspartate	D	0.01	0
154	Arginine	R	0.12	0.22
	Histidine	H	0.88	0.78
211	Arginine	R	0.94	0.99
	Glutamine	Q	0.06	0.01
222	Glutamine	Q	0.99	0.99
	Lysine	K	0.01	0.01
240	Proline	P	0.53	0.52
	Serine	S	0.47	0.48

**Table 2:** Estimates of haplotypes frequencies in the studied populations: native goat population (NGP) and crossed goat population (CGP). *n*: number of animals.

Haplotype	37	137	139	142	143	146	154	211	222	240	Frequency (%) in	
											NGP (*n* = 82)	CGP (*n* = 34)
Hp1	G	M	R	I	H	N	R	R	Q	P	43	34.5
Hp2	V	-	-	-	-	-	H	-	-	-	0	1
Hp3	-	I	-	-	-	-	-	-	-	-	4	3
Hp4	-	-	S	-	-	-	-	-	-	-	1	0
Hp5	-	-	-	M	-	-	-	-	-	-	0	4
Hp6	-	-	-	-	R	-	-	-	-	-	1	0
Hp7	-	-	-	-	-	D	-	Q	-	-	0	1
Hp8	-	-	-	-	-	-	H	-	-	-	3	9.25
Hp9	-	-	-	-	-	-	H	-	-	S	19	1
Hp10	-	-	-	-	-	-	-	Q	-	S	1	4.25
Hp11	-	-	-	-	-	-	-	-	K	S	1	1
Hp12	-	-	-	-	-	-	-	-	-	S	27	41

-: Indicates no amino acid change with respect to Hp1.

**Table 3:** Frequencies (%) of different genotypes detected in native goat (NGP) and crossed goat (CGP) populations. *n*: number of animals.

Genotypes	Frequency (%) in	
	NGP (*n* = 82)	CGP (*n* = 34)
S240S	11	15
S240P	24.5	21
P240P	19.5	15
R154H; S240S	6	2.75
H154H; S240S	6	0
R154H; S240P	14.5	12
R154H; P240P	2.5	0
H154H; P240P	0	2.75
H154H; S240P	1.25	0
R154H; R211Q; S240S	1.25	0
I142M; P240P	0	5.75
I142M; S240P	0	2.75
G37V; R154H; S240P	0	2.75
M137I; P240P	2.5	2.75
M137I; S240P	3.5	2.75
N146D; R211Q; P240P	0	2.75
Q222K; S240S	0	2.75
R211Q; S240P	0	5.75
R211Q; S240S	1.25	2.75
H143R; P240P	1.25	0
M137I; R154H; P240P	2.5	0.75
Q222K; S240P	1.25	0
R139S, P240P	1.25	0

**Table 4:** Reynolds distance (DR) estimates (above the diagonal) and pairwise F_ST_ differences (below the diagonal) among pairs of populations: native goat population (NGP) and crossed goat population (CGP).

Goat Populations		(1)	(2)
NPG	(1)	-	0.011
CGP	(2)	0.005	-

## Data Availability

The data used in this study are available on request from the corresponding author.
